# PD36 Assigning GRADE Levels To An Overview Of Reviews Using General Principles Identified From Current GRADE Guidelines

**DOI:** 10.1017/S026646232400299X

**Published:** 2025-01-07

**Authors:** Andrew Dullea, Lydia O’Sullivan, Kirsty O’Brien, Patricia Harrington, Maeve McGarry, Susan Ahern, Maeve McGarry, Kieran A. Walsh, Susan Smith, Máirín Ryan

## Abstract

**Introduction:**

Existing guidelines on overviews of reviews and umbrella reviews recommend an assessment of the certainty of evidence but provide limited guidance on how to apply GRADE to such a complex evidence synthesis. We present one approach to applying GRADE to an overview of reviews developed using general principles derived from current GRADE guidelines.

**Methods:**

The methods were developed in an iterative and exploratory fashion following discussion with 11 methodologists and health services researchers. Key principles were distilled on the five GRADE domains (risk of bias, inconsistency, imprecision, indirectness, and publication bias) from the relevant GRADE guidelines, particularly those on test accuracy.

**Results:**

A ‘general principles’ approach of applying the five domains of GRADE to an overview of reviews and arriving at an overall summary judgment for outcomes was developed. These methods were successfully applied to an overview of reviews on 18F-prostate specific membrane antigen positron emission tomography and computed tomography in the staging of patients with high-risk or recurrent prostate cancer.

**Conclusions:**

Our approach distilled key principles from relevant GRADE guidelines and allowed us to apply GRADE to a complex body of evidence. Such an approach may be of interest to other researchers working on overviews of reviews or umbrella reviews.

